# Multiple regression analyses to determine the effect of sweating rate and tattoo characteristics on sweat outcome measures during exercise

**DOI:** 10.1007/s00421-022-04989-1

**Published:** 2022-07-01

**Authors:** David M. Keyes, Shyretha D. Brown, Michelle A. King, Megan D. Engel, Matthew Ciciora-Gold, Peter John D. De Chavez, Lindsay B. Baker

**Affiliations:** 1 Gatorade Sports Science Institute, PepsiCo R&D, Barrington, IL USA; 2 Beverage Processing Science and Technology, PepsiCo R&D, Barrington, IL USA; 3 PepsiCo Data Science and Analytics, PepsiCo R&D, Barrington, IL USA

**Keywords:** Chloride, Cycling, Fitness exercise, Potassium, Running, Sodium

## Abstract

**Purpose:**

To compare local sweating rate (LSR) and local sweat sodium ([Na^+^]), chloride ([Cl^−^]), and potassium ([K^+^]) concentrations of tattooed skin and contralateral non-tattooed skin during exercise.

**Methods:**

Thirty-three recreational exercisers (17 men, 16 women) with ≥ 1 unilateral permanent tattoo on the torso/arms were tested during cycling, running, or fitness sessions (26 ± 4 °C and 54 ± 13% relative humidity). Forty-eight tattoos with a range of ink colors, ages (3 weeks to 20 years), and densities (10–100%) were included. Before exercise, the skin was cleaned with alcohol and patches (3 M Tegaderm + Pad) were placed on the tattooed and contralateral non-tattooed skin. LSR was calculated from sweat mass (0.80 ± 0.31 g), patch surface area (11.9 cm^2^), and duration (62 ± 14 min). Sweat [Na^+^], [Cl^−^], and [K^+^] were measured via ion chromatography.

**Results:**

Based on the analysis of variance results, there were no differences between tattooed and non-tattooed skin for LSR (1.16 ± 0.52 vs. 1.12 ± 0.53 mg/cm^2^/min; *p* = 0.51), sweat [Na^+^] (60.2 ± 23.5 vs. 58.5 ± 22.7 mmol/L; *p* = 0.27), sweat [Cl^−^] (52.1 ± 22.4 vs. 50.6 ± 22.0 mmol/L; *p* = 0.31), or sweat [K^+^] (5.8 ± 1.6 vs. 5.9 ± 1.4 mmol/L; *p* = 0.31). Multiple regression analyses suggested that younger tattoos were associated with higher sweat [Na^+^] (*p* = 0.045) and colorful tattoos were associated with higher sweat [Cl^−^] (*p* = 0.04) compared with contralateral non-tattooed skin. Otherwise, there were no effects of LSR or tattoo characteristics on regression models for LSR or sweat electrolyte concentrations.

**Conclusion:**

There were no effects of tattoos on LSR and sweat [K^+^] during exercise-induced sweating, but tattoo age and color had small effects on sweat [Na^+^] and sweat [Cl^−^], respectively.

**Clinical trial identifiers:**

NCT04240951 was registered on January 27, 2020 and NCT04920266 was registered on June 9, 2021.

## Introduction

Measuring local sweating rate (LSR) and electrolyte concentrations has important clinical applications in sports medicine and nutrition (Gibson and Cooke 1959; Maughan and Shirreffs 2008). This is because of the critical role that sweating plays in thermoregulatory function (Gagnon et al. 2013) and the potential impact of sweat losses on fluid/electrolyte balance (Montain et al. 2006; Shirreffs and Sawka 2011). Several studies and literature reviews have examined the effect of various methodological (e.g., regional distribution, measurement technique), host (e.g., heat acclimation, sex, body size) and external (e.g., exercise intensity, environment) factors on LSR and sweat electrolyte concentrations (Baker 2017; Baker et al. 2019; Barnes et al. 2019; Dziedzic et al. 2014; Smith and Havenith 2012, 2019). However, research regarding the impact of permanently tattooed skin on LSR and electrolyte concentrations is sparse, and results are mixed (Beliveau et al. 2020; Luetkemeier et al. 2017, 2020; Rogers et al. 2019). Establishing whether tattoos impact sweat outcomes is important since the prevalence of tattoos among physically active individuals, such as athletes (Kluger 2021), military (Armstrong et al. 2000; Laumann and Derick 2006), and tradespeople (Heywood et al. 2012), is relatively high. For example, while the prevalence of tattoos in the general population of industrialized countries is ~ 10–20% (Kluger 2015), it has been reported to be as high as 36% among military recruits (Armstrong et al. 2000), 33% in FIFA World Cup players (Mueller et al. 2020), and 53% in the National Basketball Association (Webpage 2016).

The process of tattooing involves injecting ink into the dermal layer of the skin via repeated microneedle penetration. The punctures initiate an inflammatory response, and during the healing process a portion of the injected ink becomes entrapped in the dermis forming a permanent pattern in the skin (Islam et al. 2016). Because eccrine sweat glands primarily reside in the dermis, tattooing has the potential to compromise gland function and, in turn, attenuate sweat flow rate and alter sweat composition. Indeed, a study that used pilocarpine iontophoresis to stimulate sweating, found significantly lower LSR and higher sweat sodium concentration ([Na^+^]) from tattooed skin than contralateral skin without tattoos (Luetkemeier et al. 2017). While a follow-up study by the same group also found lower LSR on tattooed skin during passive heating (Luetkemeier et al. 2020), other studies have reported no effect of tattoos on LSR or sweat [Na^+^] or potassium concentration ([K^+^]) during exercise-induced sweating (Beliveau et al. 2020; Rogers et al. 2019). Potential reasons for the mixed results may be related to the method of sweat stimulation and resultant differences in sweat flow rates, as exercise produces LSRs that are 3–5 times higher than that of pilocarpine iontophoresis and passive heating (Hjortskov et al. 1995; Taylor and Machado-Moreira 2013; Vimieiro-Gomes et al. 2005). Other factors thought to perhaps play a role include tattoo age, density, and/or ink colors (Chalmers et al. 2019).

Given the varied results to date, further research is warranted to determine the potential impact of tattoos on sweating to inform best practices for sweat testing during exercise and understand whether tattoos could have a substantial impact on overall fluid and electrolyte losses. Normal bilateral CVs for LSR and sweat electrolyte concentrations have been reported to be up to 10–16% and 6–10%, respectively (Baker et al. 2018), but on average there is no statistical difference between the left and right sides of the body in the absence of tattoos (Baker et al. 2018; Dziedzic et al. 2014; Kenefick et al. 2012). By comparison, previous passive sweating studies found a consistent 50% reduction in LSR and 60% higher sweat [Na^+^] on tattooed skin (Luetkemeier et al. 2017, 2020). If these findings held true during exercise, tattoos could confound the interpretation of LSR and sweat electrolyte concentrations. For example, if sweat [Na^+^] was overestimated by 60%, a moderate sweat [Na^+^] (e.g., 45 mmol/L) would be misclassified as high sweat [Na^+^] or salty sweat (72 mmol/L) (Baker et al. 2016).

The purpose of this study was to compare LSR and sweat [Na^+^], [Cl^−^], and [K^+^] of tattooed versus contralateral non-tattooed skin. Sweating was induced via exercise because this is the method of stimulation most relevant to athletes, laborers, and the military. A wide range of tattoo ages, ink colors, and densities were included to determine which, if any, of these tattoo characteristics impact LSR and sweat [Na^+^], [K^+^], and [Cl^−^]. Similarly, a diverse range of activities and participants were tested to include a variety of LSRs to assess the hypothesis that tattoos impact sweat outcomes only at lower sweat flow rates.

## Methods

### Participants

Thirty-five healthy, recreational exercisers, with at least one unilateral permanent tattoo on the torso or arms, agreed to participate in this study. Sweat was collected from multiple tattoos per subject where applicable, thus a total of 61 tattoos from 35 subjects were tested. However, 13 tattoos were excluded because of issues with the absorbent patch on the tattooed and/or control site delaminating from the skin or falling off completely. This issue occurred mostly during the fitness sessions (*n* = 10) and a few running sessions (*n* = 3) at the triceps (*n* = 6), wrists (*n* = 2), shoulders (*n* = 2), chest (*n* = 1), biceps (*n* = 1), and flank (*n* = 1), likely because these are areas near joints or prominent muscle contractions during exercise. Therefore, a total of 48 tattoos from 33 subjects (17 men, 16 women; 37 ± 10 years; 74.5 ± 12.7 kg) were included in the final analyses. This research was approved by the Sterling Institutional Review Board (Atlanta, GA; sterlingirb.com) and has therefore been performed in accordance with the ethical standards in the Declaration of Helsinki. Participants were informed of the experimental procedures and associated risks before providing written informed consent.

### Tattoo characteristics

Characteristics of the final 48 tattoos are presented in Table 1. Tattoos in this table are organized in ascending order from lowest to highest LSR for the corresponding non-tattooed skin. Any tattoo age, color, and density were included to determine the impact of these factors on LSR and sweat [Na^+^], [K^+^], and [Cl^−^]. Mean ± SD tattoo age and density were 7 ± 5 years and 64 ± 27%, respectively. Note that only the torso and arms were included because tattoos are most common on these regions (Laumann and Derick 2006) and because sweating rates on the legs are oftentimes too low to provide sufficient sample volume for electrolyte analyses.

**Table 1:** Tattoo characteristics

Tattoo ID number	Location	Age (years)	Color	Density (%)
1	Right shoulder	6	Black	30
2	Right bicep	3	Black	85
3	Right tricep	8	Red, blue, black	78
4	Right axillary region	5	Black	30
5	Right ventral wrist	14	Black	28
6	Right bicep	3	Pink, green, yellow, black	88
7	Right bicep	3	Black	73
8	Left bicep	3	Black, green	10
9	Left tricep	0.08	Red, black, green	100
10	Left ventral forearm	4	Black	25
11	Left ventral forearm	10	Black	23
12	Left bicep	1	Green, black	95
13	Left upper chest	14	Black	70
14	Left flank	6	Black	73
15	Left tricep	8	Black	75
16	Left tricep	7	Black	85
17	Right shoulder	3	Black	75
18	Left tricep	1	Green, black	95
19	Left ventral wrist	3	Black	10
20	Left shoulder	0.08	Red, black	100
21	Right ventral wrist	8	Black	20
22	Left bicep	5	Black	80
23	Left shoulder	14	Black	45
24	Left shoulder	8	Black, pink	80
25	Right dorsal forearm	6	Black	93
26	Left tricep	20	Black	60
27	Left dorsal wrist	2	Black	28
28	Left elbow	1	Red, blue	40
29	Left tricep	12	Red	53
30	Right scapula	15	Black, orange	83
31	Left shoulder	8	Black	83
32	Left bicep	1	Black	33
33	Left scapula	20	Pink, blue, black	65
34	Left tricep	4	Black	60
35	Left dorsal forearm	4	Black	65
36	Left shoulder	8	Black	13
37	Right scapula	6	Black	95
38	Left shoulder	0.8	Black	73
39	Left dorsal forearm	7	Black	90
40	Right ventral wrist	4	Black	90
41	Right dorsal forearm	20	Black	73
42	Left dorsal forearm	4	Black	45
43	Right scapula	2	Black	63
44	Left chest	2	Black	95
45	Right scapula	5	Black	88
46	Right lower tricep	15	Black	93
47	Left dorsal forearm	3	Black	78
48	Right scapula	9	Black	60

Tattoos are organized in ascending order from low to high local sweating rate for the contralateral non-tattooed skin

### Experimental design

Each participant completed one experimental trial and the contralateral side of the tattooed area of interest served as control. Sweat samples were collected during indoor cycling (*n* = 3), outdoor cycling (*n* = 4), outdoor instructor-led group fitness sessions (*n* = 17), or outdoor running (*n* = 9). The indoor sessions consisted of moderate-intensity cycling (153 ± 19 watts) for 1.5 h in a heated environmental chamber (32 °C and 50% relative humidity). The participants in the outdoor cycling sessions were tested during routine group training rides at a self-selected pace (23 ± 2 km/h) for ~ 1.5–2.0 h. The participants in the fitness sessions were tested during routine ~ 1-h instructor-led sessions held outdoors. For the outdoor running session, subjects ran on a closed loop at a self-selected pace (11 ± 1 km/h) for 40–60 min. The indoor trials were conducted in February–March and the outdoor sessions were completed in June–October in northeast Illinois. Heat acclimation status was not assessed, but subjects tested in June–October may have been partially to fully acclimated.

### Measurements and calculations

Before indoor cycling, participants’ nude body mass was measured to the nearest 0.01 kg (KCC300 platform and ICS439 reader; Mettler Toledo, Columbus, OH, USA). Before exercise in the field, participants were weighed to the nearest 0.05 kg (BC-350; Tanita Corporation, Arlington Heights, IL, USA) in minimal clothing (men in compression shorts and women in compression shorts and sports bra). Participants of the outdoor sessions were outfitted with a global positioning system device (Garmin Forerunner^®^ 245, Garmin International, Inc. Olathe, KS, USA) to collect heart rate, energy expenditure, duration, and distance covered (cycling and running only) during exercise. For the indoor sessions, heart rate was monitored using telemetry (H10 sensor and RS400 Reader; Polar Electro, Lake Success, NY, USA) and energy expenditure (kcal) was calculated from the cycling work rate (ACSM 2014). Subjects were allowed to drink ad libitum during indoor cycling and drink/eat ad libitum during exercise in the field. All drink bottles and food products were weighed before and after consumption to determine amounts consumed (to the nearest 0.01 g indoors; PG802, Mettler Toledo, Columbus, OH, USA and in the field to the nearest 1 g; CS2000; Ohaus, Pine Brook, NJ, USA). When necessary, urine loss during exercise was collected and weighed indoors to the nearest 0.01 g (PG-5002-S; Mettler Toledo, Columbus, OH, USA) and in the field to the nearest 1 g (CS2000; Ohaus, Pine Brook, NJ, USA). Upon completion of the exercise, participants were asked to towel dry and were weighed to the nearest 0.05 kg using the same scale and in the same manner (nude or in minimal clothing) as the pre-exercise measurement. The air temperature and relative humidity were measured with a Kestrel 5400 (Nielsen-Kellerman Co., Boothwyn, PA, USA).

Before exercise, the patch sites were shaved as needed, cleaned with alcohol, and allowed to air dry. Next, absorbent patches (Tegaderm + Pad, 3 M, St. Paul, MN) were applied to the tattooed and contralateral non-tattooed area. The correct contralateral patch site was estimated visually and by direct side-by-side comparison where possible (i.e., by bringing the right and left wrists, forearms, or triceps together). Pictures of the tattooed skin were taken before and after patch application so that investigators could subsequently estimate the density of the tattooed area and to note the colors of the tattoo ink. Tattoo density was defined as the percentage of the absorbent patch surface area that was covering tattooed skin. Two independent raters estimated tattoo density to the nearest 5% based on visual inspection of the pictures. Tattoo density values reported in Table 1 are the means of the two independent raters. The difference between raters was within ± 10% for all tattoos. Participants were also asked the age of their tattoos. Where possible (mostly on the forearms and wrists), an elastic netting (Surgilast; Derma Sciences, Princeton, NJ) was used to help ensure the patches remained adhered to the skin. During the indoor cycling and outdoor fitness sessions, investigators monitored patches regularly and removed them upon moderate saturation (based on visual inspection) to ensure sufficient sample volume for electrolyte analyses. During the outdoor cycling and running sessions, investigators met subjects at pre-designated checkpoints to check on the saturation levels of the patches and remove them if ready; otherwise, patches were removed at the end of the exercise. When ready, patches were removed from both the tattooed and contralateral non-tattooed sides (one right after the other). The absorbent pad was immediately separated from the Tegaderm using clean forceps and placed in an air-tight plastic tube (Sarstedt Salivette, Nümbrecht Germany). The tubes were sealed with Parafilm^™^, placed in a plastic bag, and transported to the GSSI laboratory for subsequent processing and analyses. Samples were stored in an ice chest (~ 10 °C) in the field and during transportation and then in a refrigerator (2–3 °C) in the laboratory. When overnight storage was needed sample analyses took place within two days of sample collection. Immediately before/during the patch removal process, we assessed the patches’ adherence to the skin for quality control purposes. Patch data were deemed invalid if the absorbent pad was visibly exposed due to Tegaderm delamination (Baker et al. 2017).

LSR (mg/cm^2^/min) was measured gravimetrically based on sweat mass absorbed in the pad (to the nearest 0.001 g using an analytical balance; Mettler Toledo Balance XS204, Columbus, OH), pad surface area (11.9 cm^2^), and patch duration on the skin. Sweat mass absorbed in the pad was calculated as post-removal patch mass minus pre-application patch mass. As mentioned above, the Tegaderm part of the patch was separated from the pad upon removal from the skin. This is done to avoid potential contamination from external sources since the Tegaderm on the topside of the patch is exposed. Thus, the Tegaderm was not included in the post-removal mass measurement. Before application, the patch mass was measured with the Tegaderm intact but was corrected by subtracting the mass of the Tegaderm alone (based on mean Tegaderm mass from several patches).

Sweat was extracted from the pad via centrifuge and analyzed in duplicate for [Na^+^], [Cl^−^], and [K^+^] via ion chromatography (Dionex ICS-6000, Thermo Fisher Scientific). Sweat data were reported using Chromeleon Software 7.2.10. Corrections to sweat [Na^+^] and [Cl^−^] for background electrolytes in the absorbent pads were made according to a previous study (Baker et al. 2018). Briefly, we have previously tested the absorbent patches for the presence of background electrolytes by pipetting known volumes of artificial sweat samples with a range of electrolyte concentrations on the pad. It was determined that background Na^+^ and Cl^−^ are present in the absorbent pads and the effect on sweat concentrations varies with the volume of sweat added to the pad. Thus, corrections to local sweat [Na^+^] and [Cl^−^] were made by subtracting the background mmol/L of electrolytes, which were calculated using the following regression equations (Baker et al. 2018): (1) background [Na^+^] = − 4.377 ln (sweat mass) + 4.300 (*r*^2^ = 0.98) and (2) background [Cl-] = − 1.602 ln (sweat mass) + 3.586 (*r*^2^ = 0.86). No corrections were needed for sweat [K^+^]. The CV of the regional absorbent patch method itself (i.e., variation introduced by background electrolytes in the pad plus analytical method variation) for sweat electrolyte concentrations is 3–5% (Baker et al. 2018). Whole-body sweating rate (WBSR) was calculated from the pre- to post-exercise change in body mass, corrected for fluid/food intake, urine loss (when applicable), and respiratory water loss and metabolic mass loss (Cheuvront and Kenefick 2017). We used 1.0 g/mL to convert mass to volume to express WBSR as L/h.

### Statistical analyses

Analyses were carried out using Graphpad Prism (Version 9.3) and Minitab 17 Statistical Software (Minitab). Visual inspection of frequency histograms and Q–Q plots as well as Shapiro–Wilk tests were conducted to assess the normality of the data. Analysis of variance (ANOVA) was used to evaluate differences between tattooed and non-tattooed skin for LSR and sweat [Na^+^], [Cl^−^], and [K^+^]. Based on a previous study (Luetkemeier et al. 2017) where sweat [Na^+^] was 69.1 ± 28.9 for tattooed skin and 42.6 ± 15.2 mmol/L for non-tattooed skin (or effect size = 1.01), the sample size required to achieve 80% power at 0.05 level of significance was *n* = 10. Subject was included in the model to account for multiple data points per subject. If the normality assumption was not satisfied, the Box-Cox transformation technique was used to identify the optimal data transformation. Raw non-transformed data are also shown in results (mean ± SD) for ease of interpretation. Bland–Altman analysis and 95% limits of agreement (LOA) were used to assess agreement between tattooed and non-tattooed skin outcomes. Sign Tests were conducted to determine if the median difference between tattooed and non-tattooed skin was significantly different from zero. Intraclass correlation (ICC, absolute agreement) and CVs (ratio of standard deviation to mean for tattoo and control) were performed to determine the reliability between tattooed and non-tattooed skin outcomes.

Multiple regression was used to assess the effect of low/high LSR and tattoo age, color, and density on tattoo versus non-tattoo differences in LSR, sweat [Na^+^], [Cl^−^], and [K^+^]. An a priori sample size calculation was not conducted for the multiple regression analyses because no previous data were available regarding the effect of tattoo color, density, or age on sweat [Na^+^] or LSR. However, the goal was to recruit and test enough subjects to include a diverse group encompassing a broad range in tattoo color (≥ 10 colorful tattoos), density (10–100%), and age (≤ 1 year to ≥ 10 years). In instances of deviation from normality (sweat [K^+^]), data were log-transformed prior to performing regression analysis. Binary classifications of tattoo age and ink density were based on the distribution of data (median values were 5 years and 73%, respectively). Tattoo age was defined as newer (≤ 5 years, *n* = 25) versus older (> 5 years, *n* = 23). Tattoo color was defined as black ink only (*n* = 36) or colorful ink (any tattoo ink color other than or in addition to black, *n* = 12). Tattoo density was defined as high density (> 70%, *n* = 26) or low/moderate density (≤ 70%, *n* = 22). Site LSR was defined as low (< 1.0 mg/cm^2^/min, *n* = 18) or high (≥ 1.0 mg/cm^2^/min, *n* = 30) based on the mean of tattoo and contralateral non-tattoo LSRs. For completeness, multiple regression with independent variables (LSR, age, and density) expressed as continuous data was also conducted. For both multiple regression analyses, all 48 individual data points were included, with the subject identification number used as a blocking factor in the model to handle multiple data points per subject. The significance level for all statistical tests was set at α = 0.05. Data are shown as means ± standard deviation (SD) unless indicated otherwise.

## Results

### Descriptive data

The duration of exercise for indoor cycling, outdoor cycling, fitness, and running was 90 ± 0 min, 99 ± 11 min, 56 ± 2 min, and 54 ± 6 min, respectively; for an overall mean of 64 ± 17 min. Heart rate and energy expenditure during exercise were 160 ± 31 bpm and 994 ± 102 kcal for indoor cycling, 139 ± 13 bpm and 725 ± 283 kcal for outdoor cycling, 152 ± 17 bpm and 684 ± 177 kcal for fitness, and 169 ± 10 bpm and 734 ± 187 kcal for running. The air temperature and relative humidity were 32 °C and 50% for indoor cycling, 25 ± 2 °C and 58 ± 17% for outdoor cycling, 27 ± 3 °C and 63 ± 4% for fitness, and 23 ± 2 °C and 37 ± 2% for running. WBSR for indoor cycling, outdoor cycling, fitness, and running were 0.85 ± 0.26 L/h, 0.58 ± 0.21 L/h, 0.99 ± 0.31 L/h, and 1.07 ± 0.31 L/h, respectively; for an overall mean of 0.95 ± 0.32 L/h. The patches were removed from the skin after 62 ± 14 min of exercise and the amount of sweat collected was 0.80 ± 0.31 g (~ 50–60% of the 1.3–1.5 g max absorbent patch capacity). This duration was chosen because of its practical relevance to sweat testing in the field-i.e., based on the duration of most training sessions and competitions in the sport. It is important to note that while prolonged application of patches to the skin may impact the local environment (potential for elevated skin temperature or hidromeiosis) (Klous et al. 2021), it would have the same effect on both sides of the body since each subject served as their own control. Thus, the duration of patch application should not affect the results of this study since it was consistent between tattooed and non-tattooed skin.

### Local sweating rate

**Fig. 1:**
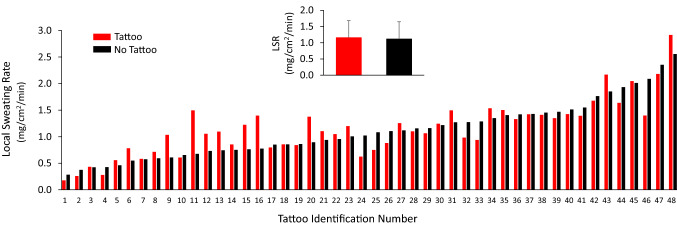
Individual data points for local sweating rate for each tattoo versus no tattoo comparison. The labels on the *x*-axis correspond with the tattoo identifier in Table 1, which are organized in ascending order from lowest to highest non-tattoo local sweating rate. Inset shows the group mean ± SD. LSR, local sweating rate. There was no statistical difference between tattoo and non-tattoo (ANOVA, *p* = 0.51)

The individual data points for LSR for each tattoo versus no tattoo comparison is shown in Fig. 1. The labels on the *x*-axis correspond with the subject and tattoo identifier in Table 1, which are organized in ascending order from lowest to highest non-tattoo LSR. The inset of Fig. 1 shows the overall group means ± SD. There was no significant difference in LSR between tattooed (1.16 ± 0.52 mg/cm^2^/min) and non-tattooed (1.12 ± 0.53 mg/cm^2^/min) skin (*p* = 0.51). The 95% LOA were − 0.42 to 0.46 mg/cm^2^/min. In addition, the median delta score between tattoo and non-tattoo LSR was not significantly different from zero (median 0.005 mg/cm^2^/min, *p* = 0.89). The tattoo versus control ICC and CV for LSR were 0.92 and 14%, respectively.

### Local sweat electrolyte concentrations

**Fig. 2:**
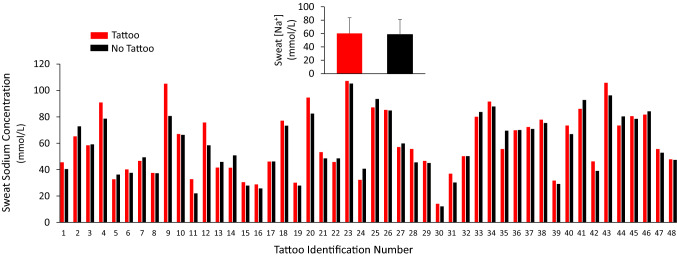
Individual data points for local sweat sodium concentration for each tattoo versus no tattoo comparison. The labels on the *x*-axis correspond with the tattoo identifier in Table 1, which are organized in ascending order from lowest to highest non-tattoo local sweating rate. Inset shows the group mean ± SD. There was no statistical difference between tattoo and non-tattoo (ANOVA, *p* = 0.27)

The individual data points for sweat [Na^+^] for each tattoo versus no tattoo comparison are shown in Fig. 2. There was no significant difference in sweat [Na^+^] between tattooed (60.2 ± 23.5 mmol/L) and non-tattooed (58.5 ± 22.7 mmol/L) skin (*p* = 0.27). The 95% LOA were − 9.9 to 11.4 mmol/L. The median delta score between tattoo and non-tattoo sweat [Na^+^] was not significantly different from zero (median = 1.7 mmol/L, *p* = 0.11). The tattoo versus control ICC and CV for LSR were 0.97 and 7%, respectively.

**Fig. 3:**
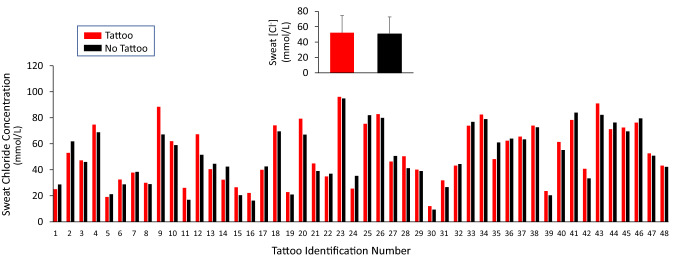
Individual data points for local sweat chloride concentration for each tattoo versus no tattoo comparison. The labels on the *x*-axis correspond with the tattoo identifier in Table 1, which are organized in ascending order from lowest to highest non-tattoo local sweating rate. Inset shows the group mean ± SD. There was no statistical difference between tattoo and non-tattoo (ANOVA, *p* = 0.31)

The individual data points for sweat [Cl^−^] for each tattoo versus no tattoo comparison are shown in Fig. 3. There was no significant difference in sweat [Cl^−^] between tattooed (52.1 ± 22.4 mmol/L) and non-tattooed (50.6 ± 22.0 mmol/L) skin (*p* = 0.31). The 95% LOA were − 9.8 to 10.8 mmol/L. The median delta score between tattoo and non-tattoo sweat [Cl^−^] was not significantly different from zero (median = 1.6 mmol/L, *p* = 0.11). The tattoo versus control ICC and CV for sweat [Cl^−^] were 0.97 and 8%, respectively.

**Fig. 4:**
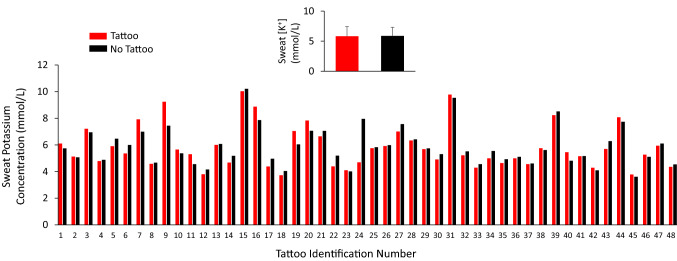
Individual data points for local sweat potassium concentration for each tattoo versus no tattoo comparison. The labels on the *x*-axis correspond with the tattoo identifier in Table 1, which are organized in ascending order from lowest to highest non-tattoo local sweating rate. Inset shows the group mean ± SD. There was no statistical difference between tattoo and non-tattoo (ANOVA, *p* = 0.31)

The individual data points for sweat [K^+^] for each tattoo versus no tattoo comparison are shown in Fig. 4. There was no significant difference in sweat [K^+^] between tattooed (5.8 ± 1.6 mmol/L) and non-tattooed (5.9 ± 1.4 mmol/L) skin (*p* = 0.31). The 95% LOA were − 1.5 to 1.3 mmol/L. The median delta score between tattoo and non-tattoo sweat [K^+^] was not significantly different from zero (median = − 0.08 mmol/L, *p* = 0.11). The tattoo versus control ICC and CV for sweat [K^+^] were 0.88 and 5%, respectively.

### Multiple regression: effect of tattoo color, age, and density and local sweating rate

**Fig. 5:**
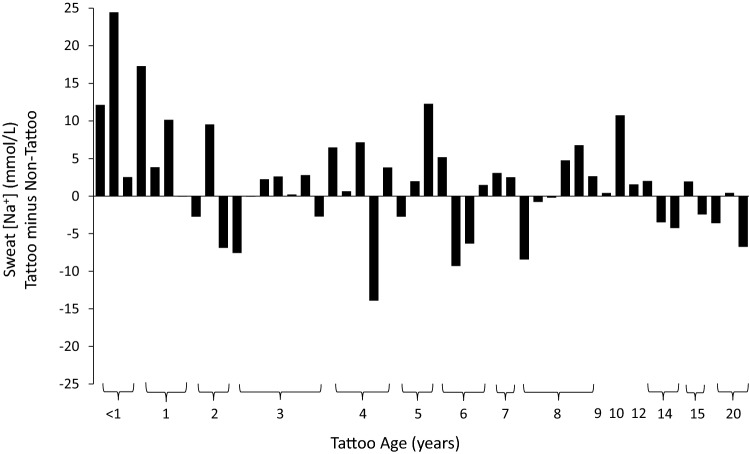
Sweat sodium concentration difference scores for each tattoo vs. non-tattoo comparison organized by tattoo age in ascending order

Table 2 shows the multiple regression analyses to determine the effect of tattoo age, ink color, density, and low/high LSR on the differences in tattooed versus non-tattooed skin for the sweat outcomes. Values in Table 2 are deltas calculated as tattooed skin minus non-tattooed skin and reported as adjusted means ± standard error. Tattoo color had a significant impact on the difference between tattooed and non-tattooed sweat [Cl^−^] (colorful ink: 4.4 ± 1.9 vs. black ink: − 0.2 ± 1.1 mmol/L, *p* = 0.04). Tattoo color, age, and density had no other significant effects on any other sweat outcomes. There were no significant effects of low/high LSR on the difference between tattooed and non-tattooed skin for sweat [Na^+^], [Cl^−^], or [K^+^]. When continuous data were used for the independent variables (where possible), the only significant result was an inverse relation between tattoo age and sweat [Na^+^] (coefficient = − 0.39, standard error coefficient = 0.19; *p* = 0.045) (Table 3), such that younger tattoos were associated with higher sweat [Na^+^] on tattooed than non-tattooed skin (Fig. 5).

**Table 2:** Multiple regression analyses with independent variables on a binary scale to determine the effect of tattoo age, ink color, density, and local sweating rate on sweat outcomes

	LSR (mg/cm^2^/min)		Sweat [Na^+^] (mmol/L)		Sweat [Cl^−^] (mmol/L)		Sweat [K^+^] (mmol/L)	
	*p *value	Adjusted mean ± SE	*p *value	Adjusted mean ± SE	*p *value	Adjusted mean ± SE	*p *value	Adjusted mean ± SE
Binary classification of independent variables								
Age								
≤ 5 years	0.81	0.03 ± 0.07	0.10	4.0 ± 1.5	0.12	3.5 ± 1.4	0.67	− 0.02 ± 0.15
> 5 years		0.06 ± 0.07		0.6 ± 1.6		0.6 ± 1.5		− 0.23 ± 0.17
Ink color								
Black	0.91	0.04 ± 0.05	0.07	0.2 ± 1.2	0.04	− 0.2 ± 1.1	0.46	0.01 ± 0.12
Colors other than or in addition to black		0.05 ± 0.09		4.5 ± 2.0		4.4 ± 1.9		− 0.25 ± 0.21
Density								
≤ 70%	0.65	0.06 ± 0.07	0.36	3.2 ± 1.7	0.48	2.7 ± 1.6	0.92	− 0.15 ± 0.17
> 70%		0.02 ± 0.06		1.4 ± 1.4		1.4 ± 1.3		− 0.09 ± 0.14
LSR								
< 1.0 mg/cm^2^/min	0.45	0.08 ± 0.08	0.41	1.4 ± 1.7	0.24	0.9 ± 1.6	0.58	− 0.10 ± 0.17
≥ 1.0 mg/cm^2^/min		0.01 ± 0.07		3.2 ± 1.5		3.3 ± 1.4		− 0.14 ± 0.15

Values are deltas calculated as tattooed skin minus non-tattooed skin

*LSR* local sweating rate, *SE* standard error

**Table 3:** *P* values from multiple regression analyses where continuous data were used for local sweating rate and tattoo age and density

	LSR *p* value	Sweat [Na^+^] *p* value	Sweat [Cl^−^] *p* value	Sweat [K^+^] *p* value
Tattoo age	0.44	0.045	0.12	0.86
Tattoo ink color	0.74	0.10	0.06	0.53
Tattoo density	0.39	0.45	0.68	0.94
LSR	––	0.91	0.85	0.36

*LSR* local sweating rate

## Discussion

This study investigated the effect of permanently tattooed skin on LSR and sweat electrolyte concentrations during exercise-induced sweating. Overall, there were no differences in LSR, sweat [Na^+^], [Cl^−^], and [K^+^] between tattooed and non-tattooed skin, which is in agreement with previous exercise studies (Beliveau et al. 2020; Rogers et al. 2019). The CVs between tattooed and non-tattooed skin were 14% for LSR and 5–8% for sweat electrolyte concentrations, which are similar to previous CVs reported for LSR (10–16%) and sweat [Na^+^], [Cl^−^], and [K^+^] (6–10%) on bilateral non-tattooed sites of the arms and torso (Baker et al. 2018). The novel aspect of the present study was the relatively large sample size and inclusion of a wide range of LSRs and tattoo ages, densities, and varied ink colors and multiple regression analyses to determine the influence that any of these factors may have had on sweat outcomes. The results suggested that tattoo ink color and tattoo age had a small, but significant impact on the difference between tattooed and non-tattooed sweat [Cl^−^] and sweat [Na^+^], respectively. LSR and tattoo color, age, and density had no other significant effects on the sweat outcomes.

The first study to investigate the effect of tattoos on the sudomotor response reported ~ 50% lower maximal and mean LSR on tattooed versus non-tattooed skin in one human volunteer after 15 min of passive heat stress (Cotton and Kuypers 1970). Surprisingly, the next study on this topic was not conducted until nearly 50 years later, where Luetkemeier et al. (2017) also found a ~ 50% lower LSR on tattooed skin when using the pilocarpine iontophoresis method to induce sweating in 10 men. In that study, sweat [Na^+^] was also significantly higher (by ~ 60%) on tattooed compared with nontattooed skin (Luetkemeier et al. 2017). The results of these initial studies would suggest that tattooing has the potential to attenuate sweat gland function, possibly attenuating reabsorption of Na^+^ in the duct as well as reducing secretory rates in the coil, at least in response to pharmacological stimulation (Luetkemeier et al. 2017) and passive heating (Cotton and Kuypers 1970; Luetkemeier et al. 2020). However, results have not been replicated in subsequent studies that used exercise to stimulate sweating. First, Rogers et al. (2019) found no effect of tattoos on LSR or sweat [Na^+^] after 20 min of exercise in 22 male and female subjects. Likewise, Beliveau et al. (2020) tested 13 men and found no effect of tattoos on LSR or sweat [Na^+^] after 60 min of cycling in the heat. The present study, which is the first to conduct multiple regression analyses on tattoo characteristics, further corroborates that tattoos have no effect on LSR and minimal, if any, impact on sweat electrolyte concentrations during exercise.

An important distinction between various methods of sweat induction is that LSR is consistently higher during exercise than pilocarpine iontophoresis or passive heating. For example, mean LSR during exercise approximates ≥ 1.0 mg/cm^2^/min (Beliveau et al. 2020; Rogers et al. 2019; Taylor and Machado-Moreira 2013) whereas LSR was usually much lower than 1.0 mg/cm^2^/min in the recent non-exercise studies (Luetkemeier et al. 2020, 2017). Thus, we included LSR as a factor in the multiple regression analysis to assess the hypothesis that tattoos only impact LSR and sweat electrolyte concentrations at low sweat flow rates. However, sweat outcomes were still unaffected by tattoos at low LSRs (< 1.0 mg/cm^2^/min) during exercise (Table 2). There were 18 patch sites with mean LSR < 1.0 mg/cm^2^/min in the present study and there were no differences between tattooed and contralateral non-tattooed skin for LSR, sweat [Na^+^], [Cl^−^], or [K^+^]. Furthermore, when continuous data were used in the model there were still no effects of LSR on sweat outcomes (Table 3).

To further elucidate potential reasons for the disparate findings across studies, we investigated how various tattoo characteristics may modify the influence of tattoos on LSR and sweat electrolyte concentrations. We considered the role of tattoo density since more ink deposition would involve more numerous skin penetrations and thus potentially greater sweat gland trauma. However, like the findings of Rogers et al. (2019), more densely tattooed skin did not impact LSR or sweat electrolyte concentrations. No previous studies have reported the ink color of the subjects’ tattoos or how this factor may have influenced LSR and sweat electrolyte concentrations. In the present study 12 of the 48 tattoos had one or more ink colors other than or in addition to black. Interestingly, colorful tattoos were associated with statistically higher sweat [Cl^−^] (*p* = 0.04) and a trend for higher sweat [Na^+^] (*p* = 0.07). However, the differences were relatively small (4–5 mmol/L), and unlikely to have practical implications with respect to whole-body electrolyte balance (Montain et al. 2006).

In previous studies the age of participants’ tattoos was ~ 2–3 years on average, ranging from a few months to a maximum around 5 years (Beliveau et al. 2020; Luetkemeier et al. 2017; Rogers et al. 2019). In the present study, there were no differences in any sweat outcome measures when comparing tattoos ≤ 5 years versus > 5 years of age. However, the multiple regression using continuous data suggested a small, but statistically significant (*p* = 0.045) effect of tattoo age on the difference between tattoo and non-tattoo sweat [Na^+^]. Specifically, there was an inverse relation, which seemed to be driven mostly by higher sweat [Na^+^] values for tattoos ≤ 1 year old. As shown in Fig. 5, six out of seven tattoos ≤ 1 year old had higher sweat [Na^+^] than non-tattooed skin. However, this does not necessarily explain the discrepancy between exercise and passive sweating studies since they both tested relatively young tattoos in the same approximate age range. Furthermore, while these results are interesting, they should be interpreted in the appropriate context. Most of these participants had high sweat [Na^+^] (see tattoo identification numbers 9, 12, 18, 20, 28, 32, and 38 in Fig. 2), as non-tattooed sweat [Na^+^] was 66.5 ± 15.0 mmol/L and tattooed sweat [Na^+^] was 76.6 ± 19.5 mmol/L. Therefore, sweat [Na^+^] was only 15% higher on tattooed skin (ranging from 0 to 30%), which is much lower than the 60% higher sweat [Na^+^] on tattooed skin found in the electrochemical study (Luetkemeier et al. 2017).

Taken together, the present study is now the third of its kind showing no or minimal effect of tattoos on LSR or sweat electrolyte concentrations during exercise-induced sweating (Beliveau et al. 2020; Rogers et al. 2019). The reason for disparate results between exercise and passive sweating studies may be related to mechanisms of sweat gland stimulation. With pilocarpine iontophoresis, eccrine sweating is induced via local cholinergic stimulation. During passive heating increases in body temperature sensed by central and skin thermoreceptors leads to stimulation of eccrine glands. By comparison, exercise-heat stress usually involves greater thermal load (metabolic heat production plus ambient heat gain), thus increasing the overall demand for evaporative cooling compared to passive heating alone, as well as contributions from non-thermal mediators of eccrine gland stimulation (central command, muscle metabo-/mechanoreceptors, and osmoreceptors) (Shibasaki and Crandall 2010).

## Conclusion

The overall results of this study suggest there are no effects of tattoos on LSR and sweat [K^+^] and marginal effects on sweat [Na^+^] and [Cl^−^] during exercise. Tattoo age and ink color had small but statistically significant effects on the difference between tattooed and non-tattooed sweat [Na^+^] and [Cl^−^], respectively. LSR and tattoo color, age, and density had no other significant effects on the sweat outcomes. These results are generally consistent with other studies reporting no effect of tattoos on LSR and sweat electrolyte concentrations during exercise-induced sweating. Moreover, these findings suggest that local sweat sampling and analyses from tattooed skin should have no impact on the practical interpretation of personalized sweat test results when conducted during exercise.

## Abbreviations

CV: Coefficient of variation
ICC: Intraclass correlation coefficient
LOA: Limits of agreement
LSR: Local sweating rate
SD: Standard deviation
SE: Standard error
[Cl^−^]: Chloride concentration
[K^+^]: Potassium concentration
[Na^+^]: Sodium concentration

## Declarations

### Data availability

All data generated or analyzed during this study are included in this published article.
